# Inaugural Lifestyle Medicine Editorial: Lifestyle medicine in the time of COVID‐19

**DOI:** 10.1002/lim2.1

**Published:** 2020-06-11

**Authors:** Fraser Birrell

**Affiliations:** ^1^ The Medical Research Council Versus Arthritis Centre for Integrated Research into Musculoskeletal Ageing Newcastle University Newcastle upon Tyne UK; ^2^ Department of Rheumatology Northumbria Healthcare NHS Foundation Trust Ashington UK

When first drafting this inaugural editorial in November 2019, events were already starting to unfold on the other side of the world, which would lead to the current pandemic, transform all of our lives and cause a still‐mounting death toll. We now know that the first case of what is now named COVID‐19 was traced back to 17 November 2019 in Wuhan, Hubei province, China and that at least 266 people were infected last year,^1^ despite initial reports suggesting the very end of December for case zero. However, this version of events was inconsistent with a case identified retrospectively from a retained swab in France taken 27 December 2019 (BBC, 2020)^2^, although this report is awaiting confirmation from the French Government. Sequencing supports a current scientific consensus that the SARS‐CoV2 coronavirus is of natural origin, with 96% homology with a bat coronavirus (RaTG13) and all six key residues from the pangolin spike receptor‐binding domain.^3^ We know the SARS virus had further outbreaks after the initial outbreak on at least four occasions at three labs in China, Taiwan, and Singapore.^4^ However, in May 2020 speculation continues about whether patterns of mobile phone activity in October 2019 indicate a “hazardous event” between 6 and 11 October causing a shutdown of the Wuhan Institute of Virology.^5^

There have been a variety of commentators, with academic leadership from the *New England Journal*,^6^ *JAMA*,^7^ and *Lancet*,^8^ with Richard Horton critical about the unheeded timely warnings about testing and personal protective equipment (PPE) requirements. Changes in UK advice on staff use of PPE in hospital outpatients in May, plus patients and other visitors and public transport users using face coverings from June have brought guidance in line with the practice of those of us informed by both the ecological evidence from countries who have coped well with the pandemic and the precautionary principle. Trish Greenhalgh has been highly influential on Twitter and in print on the revision of this guidance (Greenhalgh et al, 2020).^8^ However, some of the most useful literature has been open access and online. For example, Tomas Pueyo is a Silicon Valley online educator, who rose to international prominence with a razor‐sharp, insightful, comparative international analysis on the causes and consequences of failing to arrest spread of such an infectious and relatively deadly pathogen^10^ and the subsequent impact of relaxing control measures.^11^ As I write the first revision, these articles have been viewed 40 million and 10 million times, respectively, with a total of 60 million by the time of publication. The reasons why this was so powerful include it being a fresh perspective, an honest broker calling the politicians to account and relating, despite chagrin and shame, how quickly our health systems would and have been overwhelmed, especially in Italy, Spain, and the United States. However, social media also had an important bearing with Trish Greenhalgh and many others tweeting the link and there can be no doubt that policy has been both formed and changed as a result.

The publication in The Observer newspaper of the full Imperial College modeling paper^12^ that had informed government policy and projected around the 500 000 UK deaths and the 2.2 million US deaths without mitigation strategies certainly raised public awareness of the serious implications of inaction, even if the paper had not yet been peer‐reviewed. If published in a journal, questions raised about inconsistencies would have been in the public domain rather than in unanswered emails. However, we must have sympathy both for scientists being judged publicly on transgressing policies they have informed, as well as politicians affected by Covid‐19, whose policies might have protected the population better. The latest projections from the same team suggest 40 million deaths globally without mitigation.^13^ Another non‐peer‐reviewed paper from Imperial College from an Electrical Engineer and leader of the writing team for Right Care^14^ showed how simple second‐order modeling of death rates after effective social isolation measures were implemented could estimate much lower mortality in the range 4300‐7000 in the United Kingdom. They showed even lower rates achievable—as low as 231 deaths with aggressive testing and technologically informed contact tracing, developed for SARS and used successfully in South Korea, Taiwan, and Japan. But even at the time of writing the first draft, the accuracy of this model was uncertain and seemed overly optimistic when the NHS Medical Director was indicating the United Kingdom would “do well” to keep deaths under 20 000. Now the scale of the pandemic is clearer, we know both were overly optimistic. There have been over 7 million cases and 400,000 deaths as of early June, of which the United States has a third of cases and the highest number of recorded deaths globally, and the United Kingdom has the highest recorded deaths in Europe, now that care home deaths are included.

Similarly, public pressure for repurposing of existing drugs such as hydroxychloroquine, based on one small study of questionable design and methodology and analysis, peer‐reviewed within 24 h,^15^ resulted in France and Italy using it routinely. This led to problems completing adequately powered trials that would have otherwise provided better evidence and given time for scaling of production in order to protect supply of the drug for both those with connective tissue disease and COVID‐19, if justified. Concerns about toxicity led the WHO to pause the hydroxychloroquine arm of the global Solidarity COVID‐19 Clinical Trial after a registry analysis was published showing higher mortality from hydroxychloroquine and chloroquine with and without macrolides was published in the Lancet (Mehra et al, 2020).^16^ However, inconsistencies in the data have already led to publication of an ‘expression of concern’ (Lancet, 2020),^17^ swiftly followed by retraction (Mehra, Ruschitzka & Patel, 2020)^18^ when Surgisphere would not comply with a full independent data review. The efficacy and any major toxicity of hydroxychloroquine has been rapidly more definitely addressed by interim analysis of the ongoing RECOVERY randomised controlled trial, which has recruited 11,000 COVID‐19 patients. Due to this furore, their independent monitoring board were asked by MHRA to review the hydroxychloroquine data, which confirmed the 1542 subjects already randomised to that arm did not benefit‐ 25.7% dying compared to 23.5% deaths with usual care. While some commentators have criticised the Lancet and New England Journal, who also published another Surgisphere paper that has now been retracted (NEJM, 2020),^19^ this is actually an excellent example of the full ongoing peer review process, which does not end with publication.

In the time of COVID‐19, some of the things that we need to reconsider are as follows:

1. How important is open access?
2. How important is peer review?
3. What can we learn from COVID‐19?
4. How does that relate to existing evidence on lifestyle?

We have known for a decade that “eating a healthy diet, increasing physical activity, and avoiding tobacco use can prevent 80% of premature heart disease, 80% of type 2 diabetes cases, and 40% of cancers.”^20^, ^21^ However, the trends for these key pillars of lifestyle are disturbing: rising obesity is associated with lack of improvements in diet and activity and mortality, and the percentage of global total deaths per annum are increasing from ischemic heart disease (9.4 million deaths, 17%), diabetes mellitus (1.6 million deaths, 3%), and cancer (4.1 million deaths, 7%). Even tobacco use, which has declined substantially in most countries globally, is increasing in certain post‐Soviet Commonwealth of Independent States countries, with rates above 40% in men and attributable cardiac, respiratory, and cancer disabled life burden increasing in Eastern Europe as a whole.^22^, ^23^ There has been precious little interest in public health and scant regard for funding or implementation.

However, this pandemic has changed our approach to everything and that includes a heightened understanding of the importance of exercise—one of very few valid reasons to leave the house—nutrition, stress, and social relationships. Add in sleep and avoiding smoking plus other toxins and the six main pillars of lifestyle medicine are suddenly at the center of public consciousness, along with new insights into the scientific method, vaccine development, and clinical trials. There is substantial interest in virtual group consultations (https://bslm.org.uk/vgc/), as that seems to be an innovation whose time has come and may yet become a default in these times of self‐isolation and social distancing.

The response from the public has been exceptional and it is at times like this that we are reminded that our society is both real and functional. Examples include #ClapforCarers, #StayAtHome, #StayAtHomeAndStaySafe, and numerous examples of online exercise, mental health, and nutrition advice. Social media platforms like Twitter and Instagram have become both more important and probably kinder places than before. Positive messaging and support for the science underpinning far‐reaching lifestyle changes has been evident from role models like @DrMichaelMoseley, @DrZoeWilliams, @DoctorChrisVT, @DrRanj, and @helenlawal. There has also been a massive increase in the use of communication tools including Skype, Zoom, and Microsoft Teams (which was made available without cost to the NHS). These platforms make it possible for the British Society of Lifestyle Medicine meeting 3‐5 September 2020 to take place virtually and the Australasian Society of Lifestyle Medicine to hold a world class, international online summit on the weekend of 16‐17 May 2020, prior to their rescheduled Melbourne meeting 4‐6 December 2020. The Lifestyle Medicine community is leading by example.

## WHY IS PROGRESS SO SLOW AND HOW CAN WE MAKE A DIFFERENCE?

There have certainly been vested interests, including corporations whose profits depend on undesirable lifestyles. Governments have also not been equally proactive in making changes that may undermine revenue from “sin taxes,” making lobbying more effective. However, there have also been barriers to sharing evidence, building scientific consensus, and empowering people to make positive lifestyle choices. Importantly, there is now a groundswell: a grassroots zeal for change in the United Kingdom, where inspiration from the Australasian Society of Lifestyle Medicine (ASLM) and support from the American College of Lifestyle Medicine, combined with the passionate leadership of Rob Lawson, led to the creation of the British Society of Lifestyle Medicine (BSLM) in 2016, which has blossomed to 1100+ members in under 4 years. Campaigns like #1Change are bringing Lifestyle Medicine into the mainstream, and it is also being recognized as a potential “new medical specialty” in the British Medical Journal.^24^ Rob's subsequent appointment as President of the European Lifestyle Medicine Council (ELMC) and increasing support for other national Lifestyle Medicine Societies bodes well for wider spread.

This ever‐increasing international interest in Lifestyle Medicine is the backdrop to creation of a new open access journal “Lifestyle Medicine,” published by Wiley, which will be the official journal of ASLM, BSLM, and ELMC, with wider adoption envisaged. Your new journal needs you! We suggest you add it to your table of contents alerts, read it, submit high‐quality articles, and consider reviewing or joining the editorial board. We want to build the evidence base for lifestyle medicine, so will welcome articles from all disciplines that improve our understanding of the effects of lifestyle interventions, including exercise, nutrition, sleep, stress management, relationships, and avoidance of toxins, as well as interventions that support or enable lifestyle changes like group consultations,^25^ whether or not they are compared to drugs or other interventions.^26^ We have seen how powerful open access publication can be but would argue that rapid, robust peer review is highly desirable to ensure that decisions are made on the best evidence.

The final two questions—on COVID‐19 and the relationship to Lifestyle Medicine—are eloquently addressed in the accompanying editorial,^27^ which draws together a large amount of very recent work to explain how inflammation from ageing and chronic disease provides a unifying mechanism to explain much of the COVID‐19 risk. The positive message is that many of these risks are modifiable using lifestyle interventions.

The overarching lessons for the Lifestyle Medicine community and the world in the time of COVID‐19 are not dissimilar to those gleaned from the Gabriel Garcia Márquez masterpiece, *Love in the Time of Cholera*. We can regard Lifestyle Medicine in its myriad forms, being embraced by young and old, as a celebration of the science of healthy living and apply the lessons learned more widely. Flagging the importance of international collaboration for successful public health measures (“test, track, and trace”), supply of equipment, vaccine development, and evidence‐based treatments can only help us in achieving better health through the WHO sustainable development goals: universal health coverage, promoting healthier populations, making health systems responsive to sex and gender, and investing in data systems for health.^20^ The Lifestyle Medicine movement is ideally placed to be a key component of future medical infrastructure as we put these lessons into practice in the post‐COVID‐19 world.

## References

[1] Ma J . Coronavirus: China's first confirmed Covid‐19 case traced back to November 17. *South China Morning Post*. March 13, 2020. https://www.scmp.com/news/china/society/article/3074991/coronavirus-chinas-first-confirmed-covid-19-case-traced-back. Accessed March 29, 2020.

[2] BBC online. 5th May 2020. Coronavirus: France's first known case ‘was in December’. https://www.bbc.com/news/amp/world-europe-52526554. Accessed May 10, 2020.

[3] Andersen KG , Rambaut A , Lipkin WI , Holmes EC , Garry RF . The proximal origin of SARS‐CoV‐2. Nat Med. 2020;26:450‐455. 10.1038/s41591-020-0820-9 32284615 PMC7095063

[4] Parry J . Breaches of safety regulations are probable cause of recent SARS outbreak, WHO says. BMJ. 2004;328(7450):1222. https://doi.org.10.1136/bmj.328.7450.1222-b 10.1136/bmj.328.7450.1222-b PMC416634 15155496

[5] NBC News . Report says cellphone data suggests October shutdown at Wuhan lab, but experts are skeptical. *NBC News*. https://www.nbcnews.com/politics/national-security/report-says-cellphone-data-suggests-october-shutdown-wuhan-lab-experts-n1202716. Accessed May 10, 2020.

[6] Gates B . Responding to Covid‐19—a once‐in‐a‐century pandemic? N Engl J Med. 2020. 10.1056/NEJMp2003762 32109012

[7] Wang CJ , Ng CY , Brook RH . Response to COVID‐19 in Taiwan: big data analytics, new technology, and proactive testing. JAMA. 2020;323(14):1341‐1342. 10.1001/jama.2020.3151 32125371

[8] The Lancet . COVID‐19: learning from experience. Lancet North Am Ed. 2020. 10.1016/S0140-6736(20)30686-3 PMC7194650 32222181

[9] Greenhalgh T , Schmid MB , Czypionka T , Bassler D , Gruer L . FaceFace masks for the public during the covid‐19 /crisisFace. BMJ. 2020;369. 10.1136/bmj.m1435 32273267

[10] Pueyo T . Coronavirus: why you must act now. Politicians, community leaders and business leaders: what should you do and when. https://medium.com/@tomaspueyo/coronavirus-act-today-or-people-will-die-f4d3d9cd99ca. Accessed March 29, 2020.

[11] Pueyo T . Coronavirus: the hammer and the dance. what the next 18 months can look like, if leaders buy us time. https://medium.com/@tomaspueyo/coronavirus-the-hammer-and-the-dance-be9337092b56. Accessed March 29, 2020.

[12] Ferguson NM , Laydon D , Nedjati‐Gilani G , et al. Impact of non‐pharmaceutical interventions (NPIs) to reduce COVID‐19 mortality and healthcare demand. London, England: Imperial College; 2020. www.imperial.ac.uk/media/imperial-college/medicine/sph/ide/gida-fellowships/Imperial-College-COVID19-NPI-modelling-16-03-2020.pdf 10.1007/s11538-020-00726-x PMC7140590 32270376

[13] Walker PGT , Whittaker C , Watson O , et al. The Global Impact of COVID‐19 and Strategies for Mitigation and Suppression. London, England: Imperial College; 2020. https://www.imperial.ac.uk/media/imperial-college/medicine/sph/ide/gida-fellowships/Imperial-College-COVID19-Global-Impact-26-03-2020.pdf

[14] Pike WT , Saini V . An international comparison of the second derivative of COVID‐19 deaths after implementation of social distancing measures. MedRxiv. 2020. www.medrxiv.org/content/10.1101/2020.03.25.20041475v1.full.pdf. Accessed March 29, 2020.

[15] Gautret P , Lagier J‐C , Parola P , et al. Hydroxychloroquine and azithromycin as a treatment of COVID‐19: results of an open‐label non‐randomized clinical trial. Int J Antimicrob Agents. 10.1016/j.ijantimicag.2020.105949 PMC7779265 33408014

[16] Mehra MR , Desai SS , Ruschitzka F , Patel AN . Hydroxychloroquine or chloroquine with or without a macrolide for treatment of COVID‐19: A multinational registry analysis. Lancet. 2020. Published: May 22, 2020. 10.1016/S0140-6736(20)31180-6 PMC7255293 32450107

[17] The Lancet Editors . Expression of concern: Hydroxychloroquine or chloroquine with or without a macrolide for treatment of COVID‐19: A multinational registry analysis. Published: June 03, 2020. 10.1016/S0140-6736(20)31290-3 PMC7269709 32504543

[18] Mehra MR , Ruschitzka F , Patel AN . Retraction Hydroxychloroquine or chloroquine with or without a macrolide for treatment of COVID‐19: A multinational registry analysis. Published: June 05, 2020. 10.1016/S0140-6736(20)31324-6 PMC7274621 32511943

[19] Mehra MR , Desai SS , Kuy S , Henry TD , Patel AN Retraction: Cardiovascular Disease, Drug Therapy, and Mortality in Covid‐19. N Engl J Med. 10.1056/NEJMoa2007621 PMC7206931 32356626

[20] WHO . Unhealthy Diets and Physical Activity Factsheet. Geneva, Switzerland: World Health Organization; 2009.

[21] WHO . Disease Burden and Mortality Estimates 2000–2016. Geneva, Switzerland: World Health Organization; 2016 https://www.who.int/healthinfo/global_burden_disease/estimates/en/. Accessed March 29, 2020.

[22] WHO . European Tobacco Trends Report. Geneva, Switzerland: World Health Organization; 2019.

[23] WHO . World Health Statistics Overview 2019: Monitoring Health for the Sustainable Development Goals. Geneva, Switzerland: World Health Organization; 2019.

[24] Sayburn A . Lifestyle medicine: a new medical specialty? BMJ. 2018;363:k4442.

[25] Baqir W , Gray WK , Blair A , Haining S , Birrell F . Osteoporosis group consultations are as effective as usual care: results from a non‐inferiority randomized trial. Lifestyle Med. 2020;1:e3.

[26] Elwood PC , Longley M , Greene G , et al. Better than any pill ‐ and no side effects! Healthy lifestyles, statins and aspirin. Lifestyle Med. 2020;1:e4.

[27] Wood TR , Jóhannsson GF . Metabolic health and lifestyle medicine should be a cornerstone of future pandemic preparedness. Lifestyle Med. 2020;1:e2.

